# X-linked transcriptome dysregulation across immune cells in systemic lupus erythematosus

**DOI:** 10.1186/s13293-025-00750-3

**Published:** 2025-09-25

**Authors:** Mafalda Soares, Inês Saraiva Wemans, Paulo Caldas, Simão Teixeira da Rocha, Ana Rita Grosso

**Affiliations:** 1 Applied Molecular Biosciences Unit (UCIBIO) - Department of Life Sciences, NOVA School of Science and Technology, NOVA University Lisbon, Caparica, Portugal; 2 iBB - Institute for Bioengineering and Biosciences and Department of Bioengineering, Instituto Superior Técnico, Universidade de Lisboa, Lisbon, Portugal; 3 Associate Laboratory i4HB Institute for Health and Bioeconomy, Instituto Superior Técnico, Universidade de Lisboa, Lisbon, Portugal

## Abstract

**Background:**

Systemic lupus erythematosus (SLE) is a complex immune-mediated disease with a strong female predominance. This sex bias may be linked to the presence of two X chromosomes, which are not always adequately dosage compensated by X chromosome inactivation (XCI). Disruption in X-linked transcriptome expression may contribute to altered immune function and increased susceptibility to autoimmunity.

**Methods:**

To investigate the role of X-linked gene expression in SLE, we performed a comprehensive transcriptome analysis of 27 immune cell types from 125 female SLE patients and 66 healthy controls. We further applied a multivariate approach to integrate X-linked gene expression across all immune cell types and classify SLE patients. Additionally, we extended these models to other chromosomes and explored the correlation between autosome disease markers, including members of the XIST-interactome, and X-linked expression.

**Results:**

We observed a significant increase in X-linked gene expression in T cells, B cells and plasmablasts, while monocytes and plasmacytoid dendritic cells exhibited the opposite trend. Multivariate models based solely on X-linked expression were highly accurate and highlighted key disease-associated markers. Interestingly, autosome-based models relied on markers highly correlated with X-linked gene expression and components of the XIST-interactome, which regulates XCI. Notably, we found that *XIST* lncRNA was consistently downregulated across multiple cell types, particularly in monocytes and Th1 cells. Such downregulation correlated with increased expression of SLE-associated genes, interferon signalling, and epigenetic regulators like *KMT2D.* Further analysis revealed extensive dysregulation of the XIST-interactome in SLE, predicting X-linked transcriptome alterations in a cell-type-specific manner.

**Conclusions:**

Here, we present a comprehensive analysis of X-linked gene expression across immune cells in SLE. Our study highlights the complexity of X-linked transcriptional changes, with distinct patterns observed across both innate and adaptive immune cell types. These findings offer novel insights into the role of the X-transcriptome in sex-biased autoimmune susceptibility and may support future efforts to identify molecular targets relevant to SLE pathogenesis.

**Supplementary Information:**

The online version contains supplementary material available at 10.1186/s13293-025-00750-3.

## Abstract (plain language summary)

Systemic lupus erythematosus (SLE) is an autoimmune disease which affects far more women than men. Differences in how the X chromosome is regulated may help explain this imbalance. In this study, we analyzed the gene expression of 27 types of immune cells from 125 women with SLE and 66 healthy women. We focused on X chromosome genes and found that cells from the adaptive immune system - such as T cells, B cells and plasmablasts - showed increased expression of the genes in the X chromosome in SLE. In contrast, cells from the innate immune system, like monocytes and dendritic cells, showed decreased expression. Additionally, we used computational models to identify gene expression patterns that could distinguish SLE patients from healthy individuals. In doing so, we identified several disease-associated markers that could help better describe SLE. A key gene called *XIST*, which is a major player in the regulation of X chromosome expression, was consistently less expressed in several cell types, especially monocytes and Th1 cells. This reduction was associated with an increase in the expression of SLE-related genes and immune signalling pathways. Other genes that interact with *XIST* were also found to be disrupted in SLE. These findings highlight the differences in the regulation of the X chromosome in different cell types and give insights into the regulation of the X chromosome in SLE female patients.

**Supplementary Information:**

The online version contains supplementary material available at 10.1186/s13293-025-00750-3.

## Highlights

X-linked genes exhibit contrasting behaviour between innate and adaptive immune cells, with the X/A-ratio increasing in T cells and B cells, but decreasing in monocytes and dendritic cells.

Applying the DIABLO multivariate approach across the highest number of immune transcriptome dimensions revealed that X-linked transcriptome dysregulation is sufficient to distinguish female SLE patients from healthy controls.

Increased X/A-ratio and reduced *XIST* lncRNA expression are both correlated with elevated expression of SLE-associated genes.

X-transcriptome dysregulation is not solely explained by *XIST* lncRNA, as a significant association was found between the XIST-interactome and the X/A-ratio across immune cells.

H3K4 methyltransferases, such as *KMT2D*, were downregulated in SLE patients and strongly correlated with *XIST* lncRNA expression levels.

**Supplementary Information:**

The online version contains supplementary material available at 10.1186/s13293-025-00750-3.

## Introduction

Systemic Lupus Erythematosus (SLE) is a complex immune-mediated disease that can cause widespread inflammation and damage [1]. While SLE can affect any organ, the most common manifestations are cutaneous, musculoskeletal and hematologic. It can also cause major organ involvement, affecting the kidneys, the heart, the lungs and the central nervous system [2]. The exact mechanisms underlying SLE are not yet fully known. However, some key factors can be consistently observed: (1) break of self-tolerance due to the accumulation of apoptotic debris; (2) activation of the innate and the adaptive immune systems after the recognition of self-antigens; and (3) generation and maintenance of an extreme inflammatory response leading to tissue damage and further accumulation of apoptotic debris. This cycle shows the involvement of both innate and adaptive immune systems to create a permanent increased inflammatory state [3–6].

Innate immune cells in SLE, including monocytes, macrophages and neutrophils, exhibit impaired phagocytosis, resulting in inefficient debris clearance [3, 7]. Beyond phagocytosis, monocytes are also responsible for recognising nuclear antigens, producing cytokines, initiating the inflammatory response and recruiting other immune cells. In SLE, these functions are augmented, which is a reflection of gene expression alterations in these cells. For instance, monocytes from SLE patients show increased expression of CD40, a protein that, when it interacts with CD40L in lymphocytes, triggers their activation, proliferation and initiation of immunoglobulin switch [8]. Besides monocytes, dendritic cells are also a critical cell group in autoimmune diseases due to their role in cytokine production and lymphocyte communication [9]. Among the different types of dendritic cells, plasmacytoid dendritic cells (pDCs) are of extreme importance in SLE patients as they produce IFN𝛼, a cytokine that is prominently elevated in these patients [4, 10]. IFN𝛼 production is often a result of the activation of toll-like receptors TLR7 and TLR9, which perpetuate the activation of self-reactive T and B cells [4].

Lymphocytes have been the most extensively studied cell group in SLE due to the specificity of their response. Imbalances in the composition of T lymphocytes, namely a preponderance of T helper cells over T regulatory cells (Tregs), lead to a predominance of pro-inflammatory and B cell supporting cytokines [5, 11]. Simultaneously, the existence of fewer Tregs leads to a decrease in IL-2 cytokine production, resulting in less regulation of the immune response [11]. T cells also affect B cells, as stochastically generated autoreactive B cells are stimulated by activated B cells and start to proliferate [12]. Along with autoreactivity, B cells from SLE patients are also hyperactive across all differentiation stages, culminating in the production of long-lived autoantibody-producing plasma cells [6], worsening inflammation since these types of cells produce self-reactive antibodies for long periods.

The most significant risk factor for SLE is sex: 90% of SLE patients are women [13]. This pronounced sex bias in SLE, and other autoimmune diseases, has been attributed not only to physiological factors, like the composition of the immune system [14] and hormonal differences [15, 16], but also to genetic factors such as the X-chromosome dosage. The latter is supported by the incidence rates of SLE in patients with an abnormal number of sex chromosomes. Studies have shown that male patients with an aberrant number of X chromosome copies (47, XXY) have a similar likelihood of developing SLE as healthy females (46, XX). Similarly, female patients with an additional X chromosome (47, XXX) are twice as likely to develop SLE compared to normal females, while women with Turner’s syndrome (45, X0) have low SLE incidence [16–18]. Additionally, the overexpression of several X-linked genes, like *TLR7*, *TLR8* [19], *FOXP3* [20, 21] and *CXCR3* [22, 23] has been widely associated with SLE, especially in T and B cells. In fact, the X chromosome encloses the largest number of immune-related genes compared to other chromosomes [24]. Therefore, increased expression of X-linked genes is thought to be a key mechanism underlying sex differences in SLE.

Dosage compensation of X-linked genes between female and male mammals is ensured by X-Chromosome Inactivation (XCI) [25], a process that occurs in female cells in which one X chromosome is silenced during embryonic development through the action of the long non-coding RNA (lncRNA) *XIST*, thus creating one inactive X chromosome (Xi) and one active X chromosome (Xa) [16]. Besides *XIST*, XCI also depends on several other proteins that regulate *XIST* function and localization, termed as XIST*-*interactome [26, 27]. Despite this silencing, up to 25% of Xi genes bypass XCI and continue to be expressed from both chromosomes. These are known as escapees, and some vary between individuals, tissues, and cell types [28, 29]. For example, *TLR7* escapes XCI in a subset of immune cells, potentially influencing immune responses [30]. In SLE, the autoimmune response is exacerbated due to an increase in IFNɑ production in pDCs, which in turn activates pro-inflammatory pathways [31] and promotes the expansion of autoreactive B cells [32]. Similarly, *CD40L* and *CXCR3* in T CD4 + cells were found to be overly expressed in SLE female patients after the loss of DNA methylation upon treatment with DNMT, resulting in the overexpression of normally inactive genes [23, 33]. Although these studies suggest that XCI contributes to an autoimmune phenotype, it remains unclear the extension and impact of X-transcriptome dysregulation across the different immune lineages in SLE patients.

In this study, we applied a multivariate approach to integrate transcriptome profiles of 27 immune cell types from 125 female SLE patients and 66 healthy controls. Our analysis revealed contrasting X-linked gene expression patterns between adaptive (T and B cells) and innate (monocytes, pDCs) immune cells in SLE. Notably, we introduced the Data Integration Analysis for Biomarker Discovery using Latent cOmponents (DIABLO) [34] to integrate parallel immune transcriptome profiles. To our knowledge, this is the first time this method has been applied to integrate such profiles in the context of autoimmune diseases. Through this comprehensive approach, we developed highly accurate SLE classification models, identifying markers strongly correlated with the X-linked expression and part of the *XIST-*interactome. Notably, further analysis revealed extensive dysregulation of the *XIST* lncRNA and its interactome in SLE, predicting X-linked transcriptome alterations in a cell-type-specific manner. These findings highlight the complex and lineage-specific impact of X-transcriptome disruption on immune regulation in SLE.

## Results

### X-linked transcriptome shows contrasting modulation in adaptive versus innate immune cells in SLE

**Fig. 1:**
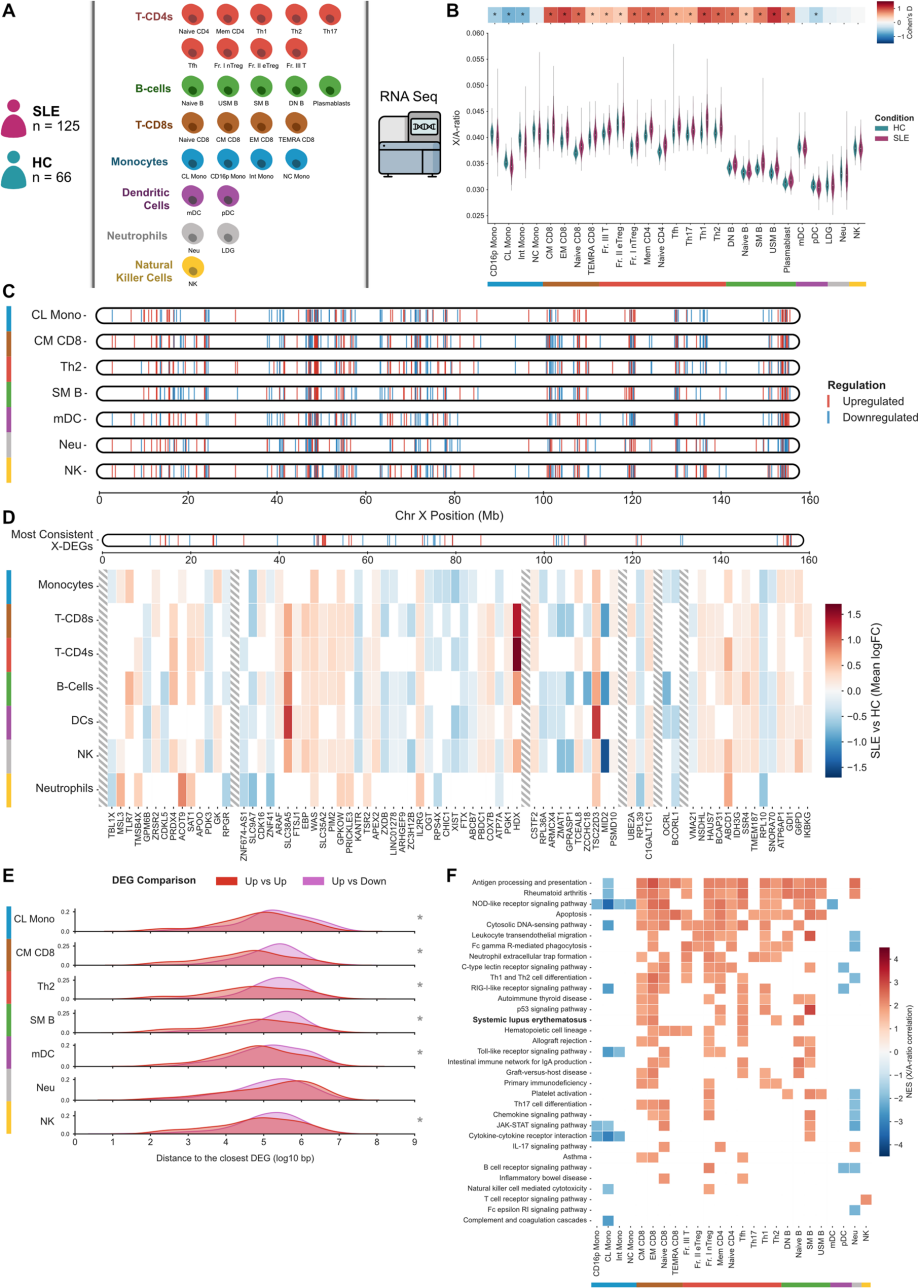
X-linked transcriptome dysregulation across immune cells in SLE patients. (**A**) Schematic representation of the transcriptome profiles for 27 cell types from 125 female SLE patients and 66 healthy controls. (**B**) Distribution of X/A-ratio values for SLE and HC samples across the different immune cell types. Top heatmap indicates the Cohen’s D effect size and statistical significance (* FDR < 0.05). (**C**) Genomic localization of up and downregulated genes in SLE along X chromosome for representative cells of each immune lineage group. (**D**) Heatmap with transcriptome alterations (mean logFC) of consistently up and downregulated genes in SLE samples along X chromosome for each immune lineage group. Columns with diagonal stripes represent large distances (> 8 Mb) between clusters of dysregulated genes. (**E**) Density plot for the genomic distance between the X-linked upregulated genes and the closest upregulated (red) or downregulated gene (pink) for representative cells of each immune lineage group (* FDR < 0.05). (**F**) Heatmap with Normalized Enrichment Scores (NES) for Gene Set Enrichment Analysis of immune-related KEGG pathways ranking genes according to the X/A-ratio correlation coefficient

To investigate potential disruptions in the X-linked transcriptome within the immune cell system of SLE, we analyzed a comprehensive transcriptome profile encompassing 27 immune cell types from 125 female SLE patients and 66 healthy controls (Fig. 1A) [35]. We first assessed the X-to-autosome (X/A-ratio) transcript ratio, defined as the sum of the raw gene counts from the X chromosome divided by the sum of the gene counts from all autosomes [36]. This ratio serves as a proxy for general upregulation of X-linked expression. We observed a significant increase in the X/A-ratio across all T cell subtypes, B cells, and plasmablasts in SLE samples (FDR < 0.05, Cohens’D > 0.37, Fig. 1B, Sup. Table 1). This pattern was corroborated by reanalyzing transcriptome profiles from other SLE cohorts focused on B cells [37] and T cells [38] (Sup. Figure 1A-B). Interestingly, we observed an inverse trend in plasmacytoid dendritic cells (pDC) and several monocyte populations (Classical (CL Mono), CD16+ (CD16p Mono), and Intermediate Monocytes (Int Mono)), where SLE patients exhibited a significantly lower X/A-ratio (FDR < 0.05, Cohens’D > 0.44, Fig. 1B). Alterations to the XCI pattern in immune cell lineages of SLE patients have already been described in B cells [19, 39], and T cells [40]. Further analysis of autosomes revealed that, apart from the X chromosome, only chromosomes 15 and 21 showed increased expression levels in SLE samples across most cell types (Sup. Figure 1 C).

To further characterize X-linked transcriptome alterations in SLE, we identified differentially expressed genes in each immune cell type by comparing SLE patients to healthy controls (Sup. Table 2). Several X-linked genes showed consistent upregulation within specific immune cell type groups and across multiple lineages (Sup. Figure 1D). Notably, these findings were consistent within each immune lineage group: monocytes, T CD8 + cells, T CD4 + cells, B cells, dendritic cells, natural killer cells and neutrophils (Fig. 1C-D). Among these genes, *TSC22D3*, *WAS*, *GPKOW*, and *IL2RG* were significantly upregulated across all immune cell types in SLE, suggesting their association is a systemic disease effect rather than a cell-type-specific effect (Sup. Figure 1E). Similarly, *SLC38A5* and *HDX* exhibited consistently elevated expression across multiple cell types. The well-known *TLR7*, previously implicated in SLE [19, 30, 41, 42], was notably upregulated in SLE patients compared to controls, in monocytes, B cells and dendritic cells. Additionally, *CXCR3*, also linked to SLE [23], consistently showed increased expression in B cells and natural killer cells (Sup. Table 2). While *TSC22D3*, *TLR7*, *CXCR3* and *IL2RG* have established links to SLE [42–45], the roles of *WAS*, *GPKOW*, *SLC38A5* and *HDX* remain largely unexplored, underscoring their potential as novel disease markers. Other genes previously associated with SLE also exhibited increased expression in specific immune cell types, namely *FOXP3* in several T cell subsets and *TLR8* in Switched Memory B cells (SM B) (Sup. Table 2). Additionally, we observed several X-linked genes that were consistently downregulated across immune cell groups, namely *PDK3*, *OGT*, *RPS4X*, *XIST*, *FTX*, *RPL39 and RPL10* (Fig. 1D, Sup. Figure 1D).

Further examination revealed that upregulated X-linked genes tended to be present in similar genomic clusters across cell types (Fig. 1C), suggesting a potential common regulatory mechanism across several genes. In fact, the distance between upregulated genes was shorter than the distance between upregulated and downregulated genes for most cell types within each cell group, with the exception of Neutrophils (Fig. 1E, Sup. Figure 1F). Although never described in immune cells, these results align with the XCI mechanism observed in human pluripotent stem cells, where repressive histone modifications encompass broad genomic regions enclosing several genes [46].

Given the disrupted X/A-ratio observed in SLE and the abundance of transcription factors and key gene regulators on the X-chromosome, we extended our analysis to the remaining genome, to identify pathways potentially impacted by X-transcriptome dysregulation. To this aim, we calculated the correlation between the X/A-ratio and expression of individual genes across the genome, and ranked the genes according to the respective correlation values for Gene Set Enrichment Analysis (GSEA). Our findings revealed significant enrichment in pathways associated with SLE and other autoimmune diseases, including: antigen processing and presentation; cytokine-cytokine receptor interaction; cytosolic DNA-sensing pathway; JAK-STAT signalling; leukocyte transendothelial migration; neutrophil extracellular trap formation; NOD-like receptor signalling pathway; rheumatoid arthritis; RIG-I-like receptor signalling pathway Toll-like receptor signalling; Th1 and Th2 cell differentiation; Th17 cell differentiation; and chemokine signalling pathway (Fig. 1F, Sup. Table 3). Additionally, we observed enrichment in the apoptosis pathway in almost all T cells and B cells subsets (Fig. 1F). Many of these pathways have been previously implicated in SLE [33, 47, 48], and our results demonstrate their association with X-transcriptome dysregulation. Furthermore, our findings further support the divergent X-transcriptome dysregulation among immune cell lineages, as reflected in the contrasting enrichment scores. Specifically, immune cells with an increased X/A-ratio in SLE, such as B cells and T cells, exhibited predominantly positive correlations with immune-related pathways. Conversely, monocytes and pDCs showed predominantly negative correlations relative to the X/A-ratio, indicating that for these cell types, lower X/A-ratios in SLE lead to upregulation of immune-related pathways.

These findings suggest that X-transcriptome dysregulation in SLE differentially impacts immune cell lineages, potentially driving distinct functional alterations in adaptive and innate immune responses.

### Integration of X-linked immune transcriptome predicts SLE and reveals genome-wide involvement

**Fig. 2:**
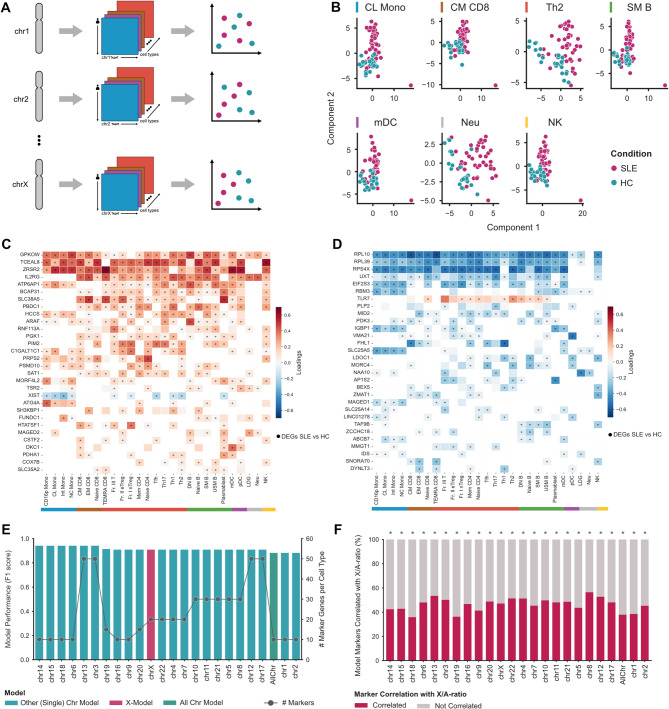
Integration of Immune Transcriptome profiles for SLE classification. (**A**) Schematic representation of DIABLO model creation, classification and evaluation (**B**) SLE and HC samples distribution according to the two components of the X-model in representative cells of each immune lineage group. **C-D**) Heatmaps with loading values for the markers selected for the first component (**C**) and second component (**D**) of the X-model for each cell type. Circles indicate differentially expressed genes in SLE versus HC. **E**) Model performance through F1 score (left axis) and number of selected makers (right axis) for each model. **F**) Percentage of marker genes significantly correlated with X/A-ratio for each single chromosome and all chromosome models

To explore the complexity of the immune system and the potential impact of X-linked genes on SLE development, we integrated X-transcriptome profiles across multiple immune cell types through Data Integration Analysis for Biomarker discovery using Latent cOmponents (DIABLO) [34]. This multivariate approach combined omics datasets to identify shared patterns and correlated features, ultimately enabling the development of a multi-cellular SLE classification model (see Methods for details) (Fig. 2A).

The X-model, based solely on X-chromosome genes, exhibited high accuracy in distinguishing SLE patients from healthy controls (Accuracy: 0.93; Precision: 1; Recall: 0.83; F1: 0.91; BER: 0.08). Using just 20 genes per cell type across the two latent components that capture the most variation within cells and correlation across all immune cells, the model effectively separated SLE patients from healthy controls (Fig. 2B, Sup. Figure 2 A, Sup. Table 4). No specific component was better in distinguishing between the two groups, but the combination of both components enhanced the overall classification of the model. Evaluation of ROC curves and AUROC indicated excellent performance across all cell types, with neutrophil subtypes (Low Density Granulocytes (LDG) and Neutrophils (Neu)) exhibiting the highest accuracy, followed by pDCs (Sup. Figure 2B). Notably, the involvement of these neutrophil subtypes and pDCs in SLE has been previously reported [9, 49, 50].

A closer look at the top contributing genes in the X-model revealed that the most prominent markers were also differentially expressed genes (DEGs) between SLE and healthy controls (Fig. 2C, Sup. Table 5). Notably, we observed distinct patterns in gene loadings signs associated with each component, indicating whether a variable contributes positively or negatively to the respective component (Fig. 2C-D). The first component, which accounted for most of the variance between sample types, mainly consisted of genes positively correlated with SLE, with exceptions such as *XIST* (Fig. 2C). In contrast, the second component was primarily composed of genes negatively associated with SLE, including exceptions like *TLR7* (Fig. 2D). Several genes previously linked to SLE emerged as markers in specific immune cell types, including *FOXP3* in T helper 2 (Th2) cells and *CXCR3* in both Th2 and Unswitched Memory B cells (USM B) (Sup. Table 5). Furthermore, several genes consistently appeared as markers across multiple cell types in the first component (*GPKOW*, *ZRSR2*, *TCEAL8*, *IL2RG* and *ATP6AP1*) and the second component (*RPL10*, *RPL39*, *RPS4X*, *UXT*, *EIF2S3*) (Fig. 2C-D, Sup. Table 5). Part of these most consistent markers were also consistently dysregulated between SLE and healthy controls, reinforcing their disease-specific relevance (Fig. 2C-D). Interestingly, *TLR7* emerged as a marker across 15 cell types, predominantly B cells and T cells (Fig. 2D). Moreover, *XIST* lncRNA was identified as a marker gene across 10 cell types, including all monocytes, most T-CD4 + cells and plasmablasts (Fig. 2C). These findings, particularly regarding *XIST* and its role in X chromosome modulation, further support the hypothesis of X dysregulation contributing to SLE pathology in a cell-context specific manner.

To assess the relevance of the X-model in SLE prediction, we compared its performance with models based on individual chromosomes and the entire genome. Interestingly, the model using all genes performed worse than models based on individual chromosomes (Fig. 2E, Sup. Table 4). Among these, models based on genes from chromosomes 14, 15, 18, or 6 showed higher performance compared to the X-model, despite its high precision and recall as measured by the F1 score (Fig. 2E). Additionally, models based on chromosomes 16, 9 and 20 had the same performance as the X-Model, but required fewer genes as markers (Fig. 2E, Sup. Table 4). Surprisingly, for most chromosomes, at least 40% of the SLE prediction markers showed significant correlation with the X/A-ratio (FDR < 0.05, |r| >0.3) (Fig. 2F, Sup. Figure 2 C, Sup. Table 4). Such results suggest that the markers identified in other chromosome-based models might be indirectly influenced by X chromosome dysregulation or vice-versa. This proportion of X/A-ratio correlated genes within the markers of the better-performing models was statistically greater than expected relative to all expressed genes (Fisher’s Exact test *p*-value < 0.05). Interestingly, several of these genes exhibited opposite correlations with the X/A-ratio in monocyte subsets and pDCs compared to T and B-cell subsets (Sup. Figure 2 C). This contrast highlights a dichotomous pattern between immune cell lineages, potentially reflecting distinct functional alterations in adaptive and innate immune responses.

Strikingly, besides *XIST* lncRNA, all our SLE classification models also identified additional markers encoding key regulators of *XIST* localization and function in XCI, collectively known as the XIST-interactome [26]. Interestingly, 23 out of the 107 components of the XIST-interactome emerged as markers within their respective chromosome-specific model (Sup. Figure 2D, Sup. Table 5).

These findings highlighted the pivotal role of X-transcriptome dysregulation in SLE across different immune cell types, suggesting a complex involvement of X-linked gene dosage mechanisms in disease pathogenesis, potentially shaping broader immune regulatory networks across the genome.

### XIST downregulation is associated with dysregulation of SLE-linked genes and epigenetic modifiers

**Fig. 3:**
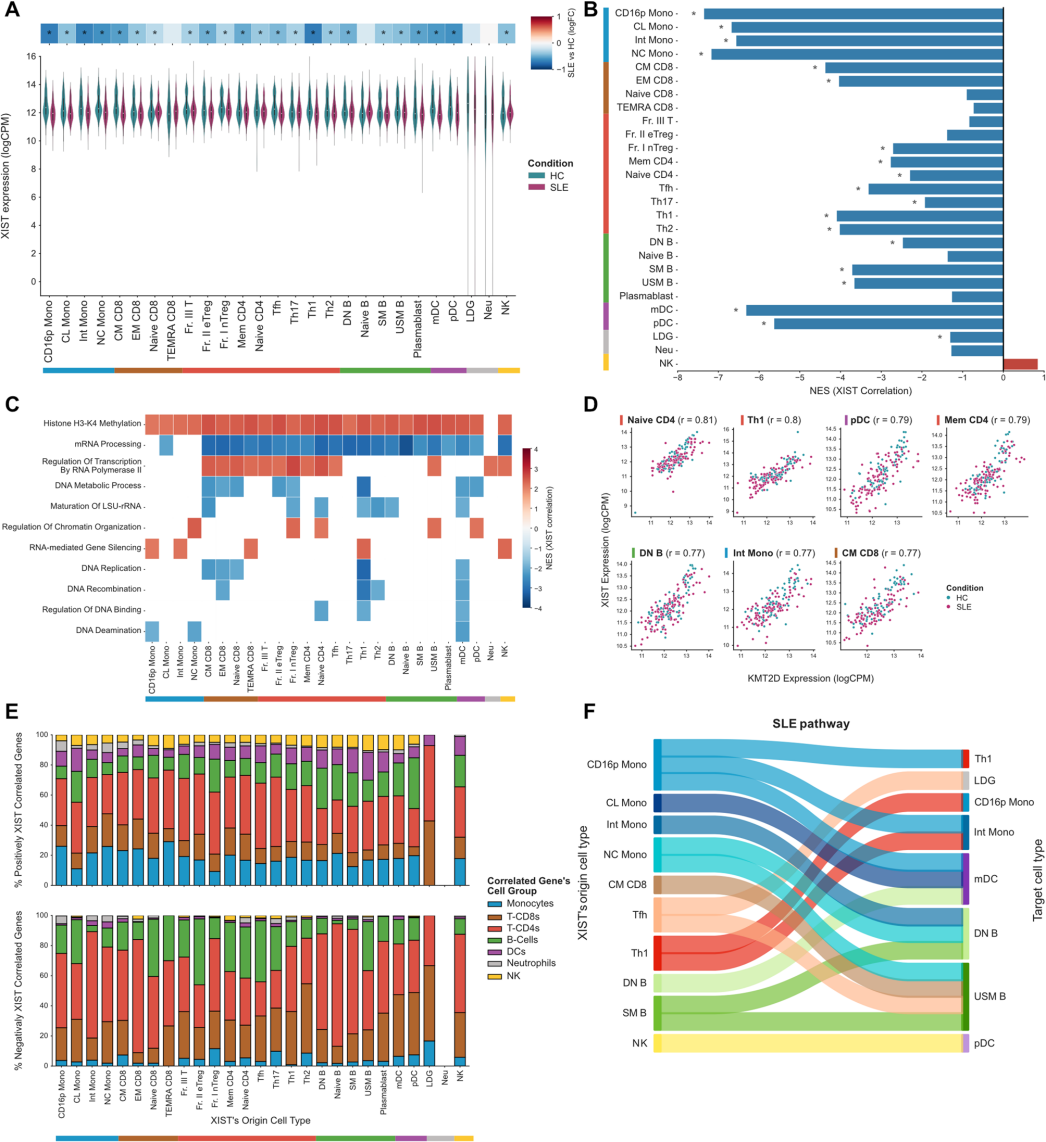
*XIST* lncRNA dysregulation across immune cells in SLE patients. (**A**) Distribution of *XIST* expression levels for SLE and HC samples across the different immune cell types. Top heatmap indicates the fold-change effect size (logFC) and statistical significance (* FDR < 0.05). (**B**) Normalized Enrichment Scores (NES) for Gene Set Enrichment Analysis of IFN-signatures ranking genes according to the *XIST* correlation coefficient. (**C**) Heatmap with Normalized Enrichment Scores (NES) for Gene Set Enrichment Analysis of GO terms for Biological Processes ranking genes according to the *XIST* correlation coefficient (FDR < 0.05). (**D**) Correlation plots and coefficients for the cell types with the strongest correlation between *XIST* and *KMT2D* expression levels. (**E**) Percentage of the significant intercellular positive (top plot) and negative (bottom plot) correlations (FDR < 0.05 and |r| >0.5) between *XIST* expression levels of each cell type (x-axis) and all other genes in other immune lineages (bar colors). (**F**) Sankey plot with the intercellular correlations where *XIST* expression in the origin cell type is correlated with the expression of SLE-associated genes in the target cell type (FDR < 0.01)

Leveraging the key role of *XIST* lncRNA in XCI and its presence in the SLE classification models, we further investigated *XIST* dysregulation and its potential impact on immune cells. Notably, comparing SLE with healthy controls, *XIST* was consistently downregulated across multiple cell types, with the most pronounced decrease observed in Th1 cells, monocytes, and dendritic cells (Fig. 3A). Other SLE cohorts were used for validation and presented dissimilar trends amongst them with no statistical significance (Sup. Figure 3A-B). Previous studies have reported mislocalization of *XIST* in B cells [39] and T cells [40] of SLE patients, without concurrent alterations at the gene expression level.

To further explore the link between the X-transcriptome dysregulation and *XIST* in SLE, we assessed the correlation between both X/A-ratio and *XIST* lncRNA expression levels for each cell type (Sup. Table 6). Surprisingly, *XIST* lncRNA showed a significant but weaker positive correlation with X/A-ratio specifically in monocyte subsets (CD16p Mono, CL Mono and Int Mono) (Sup. Figure 3 C). In contrast, other cell types, like T-CD4s, particularly T helper 1 (Th1) cells, showed the expected negative correlations (Sup. Figure 3 C).

To further explore the implications of this dysregulation in immune cells, we performed GSEA of *XIST*-correlated genes (Sup. Table 7). Similarly to pathways associated with X/A-ratio, we found a significant enrichment in immune-related signalling, including antigen processing and presentation, cytokine-cytokine receptor interaction, JAK-STAT signalling, and Toll-like receptor signalling (Sup. Figure 3D, Sup. Table 8). Notably, most of these pathways were enriched for genes negatively correlated with *XIST*, indicating that *XIST* downregulation in SLE is linked to increased expression of these immune-related genes. Specifically, the annotated SLE pathway was significantly enriched in negatively *XIST-*correlated genes for all monocytes subsets, Th1 cells, and myeloid dendritic cells (mDCs) (Sup. Figure 3D), highlighting a strong link between reduced *XIST* expression and SLE pathology.

Given that SLE is characterized by an activation of the interferon (IFN) system [51], we also assessed the enrichment of IFN-signatures [52] in *XIST-*correlated genes. Indeed, we found a significant association between reduced *XIST* expression and increased expression of IFN-related genes across several cell types, as seen by the enrichment (NES < 0) of these genes in *XIST*-correlated genes (Fig. 3B). Strikingly, 96 out of 100 IFN-related genes correlated with *XIST* in at least one immune cell type (FDR < 0.05) (Sup. Figure 3E, Sup. Table 7). The strongest associations were observed in monocytes and dendritic cells (Fig. 3B), highlighting cell-specific functions and reinforcing the connection between *XIST* dysregulation and SLE-associated pathways.

In addition to immune-linked pathways, *XIST*-correlated genes were also enriched in genome and gene expression regulation processes (Fig. 3C, Sup. Table 8). Interestingly, genes involved in DNA replication and mRNA processing showed a negative correlation with *XIST* expression, suggesting that reduced *XIST* expression in SLE activates these processes. Conversely, genes related to histone lysine methylation and transcription regulation were positively correlated with *XIST* expression, indicating potential downregulation with reduced *XIST* expression. Notably, three out of the top 10 *XIST*-correlated genes were histone lysine methyltransferases of H3K4 (*KMT2D*, *KMT2A*, *KMT2C*) and were all positively correlated with *XIST*. Similar correlations were found in the additional SLE cohorts (Sup. Figure 3F-G). *KMT2D* displayed a particularly strong positive correlation with *XIST* expression across most immune cells (*r* > 0.7, FDR < 0.00005, Fig. 3D, Sup. Table 7), and was consistently downregulated in SLE relative to healthy controls (Sup. Figure 3H). This downregulation was also observed in T cells from the validation cohort, but not in B cells, where there was no statistically significant difference (Sup. Figure 3I-J). Interestingly, *KMT2D* also emerged as a marker in our SLE-classification model based on chromosome 12 genes, for 12 immune cell types, including: monocytes (CL Mono and Int Mono), all B cells subsets, all dendritic cells, all neutrophils, and natural killer cells (Sup. Table 5). *KMT2D*, also known as *MLL2*, is responsible for H3K4 methylation, a histone modification linked to active transcription [53]. Although the precise mechanism remains unclear, our findings suggest a coordinated downregulation of *XIST* and *KMT2D* in SLE, potentially affecting transcription regulation through histone modifications.

Given the strong evidence linking *XIST* dysregulation to immune responses in SLE, we further explored how reduced *XIST* expression might influence cell-cell interactions. To this end, we performed a correlation analysis between *XIST* expression in each cell type and all genes in other cell types. Remarkably, *XIST* expression showed a significant positive correlation with several genes in other immune cell types (FDR < 0.05, |r| > 0.5, Sup. Figure 3K, Sup. Table 9). In contrast, negative correlations were predominantly observed for *XIST* expression in T CD8 + cells (Central Memory CD8 (CM CD8)), T-CD4 + cells (Fraction I natural regulatory T cells (Fr I nTreg)) and plasmablasts. Further analysis revealed that *XIST* exhibited the strongest positive intercellular correlations with genes from monocytes and dendritic cells, whereas negative correlations were more prevalent with genes from B and T-cell subsets (Fig. 3E). These results suggest that *XIST* dysregulation may have distinct effects across immune cell groups, potentially shaping interactions differently within the innate and adaptive immune systems. To investigate the pathways involved in these cell-to-cell interactions, we performed GSEA based on the *XIST*-intercellular correlation profiles. As expected, multiple cell pairs showed *XIST*-correlated genes enriched in immune-related pathways, including antigen processing and presentation and primary immunodeficiency (Sup. Table 10). Most enrichment of immune pathways results from correlations with *XIST* from Th1 cells or monocytes. Notably, several cell pairs showed *XIST*-correlated genes linked to the SLE pathway, suggesting widespread cellular interactions in SLE (Fig. 3F, Sup. Figure 3L, Sup. Table 10). Once again, monocytes stood out, with *XIST* expression leading to the enrichment of SLE pathway genes across multiple cell types. Moreover, *XIST* expression in Th1 and mDC were also linked to SLE pathway genes in other cell types. Additionally, we could also observe that *XIST* expression in monocytes, Th1 and dendritic cells was highly linked to the expression of IFN-related genes in every cell group (Sup. Figure 3M).

Hence, we could depict widespread transcriptomic changes associated with reduced *XIST* expression in SLE, linked to increased expression of SLE-associated genes and downregulation of specific epigenetic modifiers, such as H3K4 methyltransferases.

### XIST-interactome is dysregulated across all immune cell types in SLE

**Fig. 4:**
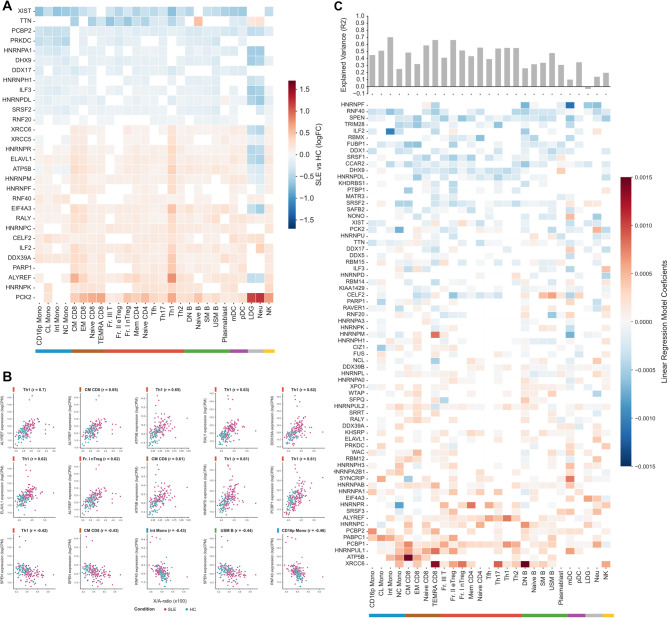
*XIST*-interactome dysregulation across immune cells in SLE patients. (**A**) Heatmap with Transcriptome alterations (logFC) of XIST-interactome genes in SLE samples for each immune cell type (FDR < 0.05). White indicates no statistically significant difference. (**B**) Correlation plots and coefficients for the cell types with the strongest correlation between X/A-ratio and gene expression levels of the *XIST*-interactome. (**C**) Heatmap with regression models coefficients associated with X/A-ratio for each cell type. The top barplot indicates the explained variance (R2) of each cell-specific model

Since XCI relies not only on *XIST* but also on the *XIST*-interactome for proper function and localization, we further examined their expression patterns across multiple immune cell types in SLE. Our analyses revealed consistent alterations in several *XIST*-interactome genes [26] across these cell types in SLE (Fig. 4A, Sup. Table 11). The most consistently dysregulated genes exhibited uniform expression patterns, being either upregulated or downregulated across cell types, with neutrophils and Naive B cells as the notable exceptions. Interestingly, these same cell types also stood out as those where *XIST* is not significantly downregulated (Fig. 3A). *XIST* showed the strongest downregulation across cell types, while, of all *XIST*-interactome genes, *PCK2* displayed the most pronounced increase in expression (Fig. 4A).

To further explore the link between the XCI and *XIST*-interactome dysregulation in SLE, we assessed the correlation between both X/A-ratio and the 107 interactome genes for each cell type. Most *XIST*-interactome genes were correlated with the X/A-ratio (Sup. Figure 4A). This result was partially replicated in the T cell validation cohort, but not in the B cell validation cohort (Sup. Figure 4B). The strongest correlations were observed for *ALYREF*, *ATP5B*, *RALY* and *DDX39A* (Fig. 4B, Sup. Table 6). Notably, *TRIM28*, a reported B cell-specific *XIST* co-factor [26], was positively correlated with the X/A-ratio in specific monocytes and T cells subtypes, but did not show a significant correlation in B cells (Sup. Figure 4A). Only a few genes exhibited consistently negative correlations with X/A-ratio across the different cell types, namely *SPEN*, *RNF40*, *TTN*, *and DDX17* (Fig. 4B, Sup. Figure 4A, Sup. Table 6). Indeed, some *XIST*-interactome genes, such as *HNRNPM*, *DDX39A*, *ILF2*, and *HNRNPR*, displayed divergent associations with the X/A-ratio, forming distinct patterns between two immune cell groups: monocytes and dendritic cells *versus* T cells, B cells (Sup. Figure 4A). These findings aligned with observed heterogeneity in X-transcriptome dysregulation across immune cell lineages (Fig. 1B). This suggests that the X-transcriptome dysregulation in SLE may not solely depend on *XIST* expression levels, but also on cell-specific *XIST*-interactome components that influence its function and localization.

Therefore, we next sought to model the X/A-ratio through expression levels of *XIST*-interactome players differentially expressed in SLE. Our specific regression models could explain at least 50% of the X/A-ratio variance observed for monocytes subsets (CL Mono and Int Mono), T CD8 + cells (Naive CD8 and Terminally Differentiated Effector Memory CD8 (TEMRA CD8)) and several T-CD4 + cells (Fr. I T nTreg, Fraction II efector regulatory T cells (Fr. II eTreg), Naive CD4, Th17, Th1, Th2), but it was not effective in LDGs and mDCs (Fig. 4C, Sup. Table 12). Again, *SPEN* and *RNF40* showed consistently negative associations with the X/A-ratio, while *ATP5B*, *ALYREF* and *XRCC6* were consistently positively associated with the X/A-ratio. Notably, the genes selected with the greatest weight to predict X/A-ratio differed between immune cell lineages. For example, in monocytes, the strongest predictors were *ILF2*, *HNRNPC and PABPC1*, whereas in T cells, X-transcriptome dysregulation was best predicted by *XRCC5*, *HNRNPM*, *CELF2*, *and DHX9* (Fig. 4C). Curiously, *XIST* expression itself was found to contribute to the prediction of X-transcriptome dysregulation in select cell types, exerting both positive and negative effects, with its most substantial impact observed in Naive CD4 cells. These findings reinforce that *XIST* lncRNA expression alone may not be sufficient for proper XCI, as it also relies on the presence of key *XIST*-interactome components. In contrast, *SPEN and RNF40* known to encode for proteins interacting with the silencing mode of *XIST*, the Repeat A [27, 54, 55], have a clearer negative relationship with X/A-ratio, suggesting they might exert a more important role in the maintenance of XCI in immune cells.

Collectively, these results highlight the complex interplay between *XIST* lncRNA, its interactome, and X-transcriptome regulation in SLE, indicating distinct modulation patterns in adaptive versus innate immune cells within the context of SLE.

## Discussion

The relationship between the X chromosome and autoimmune diseases, particularly SLE, has been extensively studied, with the XCI process being a major focus of current research [30, 39]. Here, we provide compelling evidence of X-linked transcriptome signature across multiple immune cell types in SLE.

Our analysis leveraged a comprehensive transcriptome profile encompassing 27 immune cell types from 125 female SLE patients and 66 healthy controls [35]. This large, cell-specific dataset allowed us to address both cellular and individual heterogeneity in SLE and develop robust classification models. We corroborated our findings using smaller cohorts where possible [37, 38]. Notably, most of our results were supported in the T cell validation cohort [38], but not in the B cell cohort [37]. Given the complexity of the SLE disorder, discrepancies in the results may be attributed to differences in sample size, disease activity, degree of cell-type resolution, and population differences across datasets. We acknowledge certain limitations of our work, including data access restrictions that prevented us from assessing the extent of disease activity of SLE patients and other relevant factors, such as allelic expression. Additionally, our study was conducted using data regarding an East Asian population that may exhibit differences from other populations of different ethnicities.

A key finding of this study was the significant increase in the X/A-ratio in T cells, B cells, and plasmablasts from SLE patients, whereas monocytes and plasmacytoid dendritic cells exhibited a decrease. This differential regulation suggests that changes in X-linked gene regulation in SLE are not uniform but vary across immune lineages, potentially influencing adaptive and innate immune responses differently. Previous studies have implicated XCI disruption in B cells [39] and T cells [40]. Our data gives a more complete view of the complexity of X-linked gene expression across multiple immune subsets. Furthermore, our transcriptome analysis identified groups of X-linked genes consistently up- or downregulated across multiple cell lineages, including several with well-established roles in SLE, like *TLR7* and *CXCR3* [23, 42, 45, 56]. Previous comparisons have shown a balance of upregulated and downregulated X-linked genes in T cells [40], with B cells displaying a predominance of downregulated X-linked genes [39]. Our analysis also revealed that upregulated genes tend to cluster in close genomic proximity, suggesting localized disruptions in repressive histone modifications associated with the XCI mechanism. Indeed, a shift from a repressive to an active epigenetic profile was observed in several X-linked genes regions upon *XIST* depletion [26]. However, a comprehensive allelic-specific expression analysis would be needed to determine whether this dysregulation originates from the inactive X or other regulatory mechanisms.

Our integrative analysis using DIABLO demonstrated that an X-linked gene-based classification model effectively distinguished SLE patients from healthy controls with high accuracy. Key genes such as *GPKOW*, *TCEAL8*, *ZRSR2*, *RPL10*, *RPL39*, *RPSX4* emerged as significant markers across multiple immune cell types. In contrast, genes like *TLR7*, *CXCR3* and *FOXP3*, previously linked to SLE [20, 23, 42], were more specifically associated with T and B cells subsets. Surprisingly, neutrophils subsets stood out as among the best-performing cell types in the X-based model. Neutrophils are known to exhibit defective phagocytosis, leading to the accumulation of immune complexes and nuclear material post-apoptosis in SLE[3]. Additionally, neutrophils isolated from SLE patients exhibit a distinct functional phenotype [49], and LDGs, a neutrophil subset, have been associated with SLE disease activity [50]. Hence, the X-model effectively captured cell-specific SLE characteristics while using only X-linked genes.

Interestingly, classification models based on individual autosomes achieved high accuracy in distinguishing SLE, with their gene features significantly correlating with the X/A-ratio. Thus, our study clearly shows that X-linked dysregulation may exert a disproportionate impact on SLE pathology. More importantly, to our knowledge, this study presents the first use of the DIABLO multivariate integrative approach to analyze parallel, heterogeneous immune transcriptome profiles in autoimmunity, incorporating the highest number of dimensions reported to date. Obviously, the biological relevance of machine learning models extensively relies on external validation across independent cohorts to prevent overfitting and ensure robust conclusions. However, since our model integrates expression data from 27 immune cell types, external validation presents a challenge due to the limited availability of comparable cell-specific transcription datasets from SLE patients. Future studies incorporating diverse cohorts with similarly detailed immune profiling will be crucial to validate these findings and establish X-linked genes as reliable biomarkers for SLE.

Notably, our classification models depicted *XIST* lncRNA and several *XIST*-interactome genes as markers of SLE. *XIST* was consistently downregulated across multiple cell types in SLE, which was not fully validated in the additional cohorts presented, and contrasts with previous reports describing its upregulation [57, 58]. The larger sample size and cell-type resolution of our study provides greater confidence in detecting this downregulation, as they help mitigate some of the variability of *XIST* in SLE observerd in other studies. Nonetheless, other clinical and demographical factors, such as disease activity and ethnicity, may contribute to differences in *XIST* expression across studies. Interestingly, *XIST* downregulation significantly correlated with the upregulation of SLE-linked genes, particularly those involved in IFN-signaling. Given the established role of IFN pathways in SLE, our findings suggest that reduced *XIST* expression may be a critical upstream regulator of inflammatory gene expression in SLE. Supporting this, previous studies in mice have shown that *Xist* knockdown in B cells leads to biallelic expression of normally silenced genes and development of an autoimmune phenotype [59], along with hallmark features of human SLE [60]. Furthermore, we observed a strong correlation between *XIST* levels and regulatory mechanisms such as histone modification and splicing. Recent work has shown that splicing is affected in SLE [61]. Moreover, the strongest *XIST-*correlated gene was *KMT2D*, followed closely by *KMT2A* and *KMT2C*, all of which encode H3K4 methyltransferases known to promote transcriptional activation [53]. Although the link between *KMT2D and SLE* remains largely unexplored, *KMT2D* loss has been implicated in the autoimmune phenotype observed in Kabuki syndrome [62]. Notably, previous studies have reported altered patterns of H3K4 methylation in SLE patients, particularly in monocytes, in overexpressed genes [63, 64]. The coordinated downregulation of *XIST* and *KMT2D* in SLE suggests a potential mechanistic link between XCI disruption and global transcriptional dysregulation possibly mediated by epigenetic changes.

Our correlation analysis across immune cell types also revealed potential intercellular regulatory interactions linked to *XIST* expression. Notably, monocytes and Th1 stood out, with their *XIST* expression correlating with the enrichment of SLE-related pathways and IFN-signatures in other cell types. These findings may suggest the presence of a positive feedback loop, previously described in rheumatoid arthritis, in which monocytes activate T-helper cells via pro-inflammatory cytokines like IL-12 and self-antigen presentation, while T-helper cells promote monocyte proliferation through IFNγ production [65]. *XIST* underexpression may contribute to this loop by influencing both self-antigen recognition and IFN-I production. Dendritic cells also demonstrated significant associations between *XIST* expression and SLE-related gene expression across multiple cell types. Given their critical role in antigen presentation to both T and B lymphocytes, dysregulated gene expression in dendritic cells could have systemic effects, disrupting both innate and adaptive immune responses. Although we could not fully elucidate the mechanisms driving these potential cell-to-cell interactions, recent studies have proposed novel immunogenic roles for *XIST*. One study identified *XIST* lncRNA as a potential TLR7 ligand, detectable in extracellular vesicles from dying cells, and capable of triggering autoimmune responses, at least in vitro [57]. Other studies suggest that *XIST* and its interactome may be inherently immunogenic in mice [66] and in SLE patients, particularly in neutrophils [67]. While we could not support this hypothesis in our dataset, we did observe distinct expression patterns of the *XIST*-interactome in neutrophils.

Finally, our findings underscore the association of the *XIST*-interactome with SLE, where multiple *XIST*-associated genes exhibited dysregulated expression across immune cell types. The strong correlation between *XIST*-interactome gene expression and the X/A-ratio suggests that X-linked transcriptional alterations in SLE extend beyond *XIST* levels, involving broader regulatory networks that may vary across cell lineages. Notably, monocyte subsets and pDC showed contrasting correlations compared to T and B cells, reinforcing the dichotomous pattern observed among immune cell groups. Of note, *SPEN* and *RNF40*, encoding proteins binding to the Repeat A, the silencing mode of *XIST* RNA [27, 54, 55] stand as genes with the *XIST*-interactome that correlate negatively with the X/A-ratio, suggesting they might have an impact on XCI maintenance in immune cells with potential relevance for SLE. Moreover, through linear regression modelling, we demonstrated that *XIST*-interactome genes could predict X/A-ratio variations with variable degrees of success, which may be linked to the cell-specific composition of the *XIST*-interactome [26]. Nevertheless, the success of these models reinforces the potential role of the *XIST*-interactome in shaping immune dysregulation in SLE. In fact, previous works have reported aberrant expression of *XIST*-interactome genes and *XIST* RNA mislocalization in B cells and T cells of SLE patients [39, 40].

In conclusion, our study provides strong evidence of X-linked transcriptome dysregulation across several immune cell types in SLE. Notably, the dualistic pattern between monocyte subsets and plasmacytoid dendritic cells versus T and B cells suggests that X-linked transcriptional alterations may have different functional effects in the innate and adaptive immune systems. Importantly, we observed that *XIST* downregulation was seen across different immune cell types anti-correlating with X/A-ratio in B and T cells, and correlating positively with innate immune cell types. This suggests that *XIST* expression alone may not be the main disruptor of X chromosome expression in SLE, highlighting the potential involvement of other mechanisms, perhaps involving *XIST* partners, namely the ones associated with its silencing module. Future studies focusing on the mechanistic links between *XIST* lncRNA, *XIST*-interactome and immune regulatory pathways may offer novel insights into therapeutic targets for SLE.

## Materials and methods

### Transcriptome profiles of SLE patients and healthy controls

Transcriptome profiles from Nakano et al. 2022 [35] were reanalyzed and were considered the main cohort as it was the most comprehensive cohort in terms of cell type diversity and sample size. The gene read counts from RNA-Seq (HiSeq 2500 Illumina) and clinical information were obtained through a request to the NBDC Human Database (JGAD000603). The transcriptome profiles enclosed bulk-sequencing of pre-isolated 27 immune cell types namely: T CD4 + cells – Naive CD4+, Memory CD4+ (Mem CD4), T helper 1 (Th1), T helper 2 (Th2), T helper 17 (Th17), T follicular helper (Tfh), fraction I natural regulatory T cells (Fr. I nTreg), fraction II effector regulatory T cells (Fr. II eTreg), fraction III T cells (Fr. III T) – T CD8 + cells – Naive CD8+, Central Memory CD8 + T cells (CM CD8), Effector Memory CD8 + T cells (EM CD8), Terminally Differentiated Effector Memory CD8 + T cells (TEMRA CD8) – B cells – Naive B, Unswitched Memory B cells (USM B), Switched Memory B cells (SM B), Double Negative B cells (DN B) and plasmablasts – Monocytes – Classical Monocytes (CL Mono), CD16 + Monocytes (CD16p Mono), Intermediate Monocytes (Int Mono), Non-Classical Monocytes (NC Mono) - Dendritic cells – myeloid Dendritic Cell (mDC), plasmacytoid Dendritic Cells (pDC) - Neutrophils (Neu) – Neu and Low-Density Granulocytes (LDG) - and Natural Killer cells (NKs). In total, 125 SLE female patients and 66 female HC patients with Eastern-Asian ancestry were analyzed (Fig. 1A). Some patients in the main cohort were undergoing treatment with belimumab (BLM). Samples from these patients were recovered twice, once before BLM treatment and another time after six months of BLM treatment. To ensure all patients included in this study were assessed in as similar conditions as possible, all post-treatment samples were discarded.

Two additional validation cohorts focusing on female patients were reanalyzed to corroborate the main findings in B-cell and T cells. Summarising, B-cell subsets enclosed activated naive B cells (aN), resting naive B cells (rN), transitional 3 B cells (T3), SM B, DN B and, antibody secreting cells (ASC) from 9 SLE patients and 12 HC with African-American ancestry (GSE118256) [37]. T cells transcriptome profiles included only Naive CD4 from 13 SLE patients and 4 HC patients (PRJNA293549) [38]. Data description and further details are summarized in Sup. Table 13.

### Transcriptome data processing

Expression data for the main SLE cohort was provided in raw counts and converted to Counts per Million (CPM) and logarithmically transformed to logCPMs.

For the validation cohorts, gene read counts were obtained after alignment to the reference genome (GRCh38 assembly; release 44, GRCh38.p14) using STAR (v2.7.10b) with options -quantMode GeneCounts and -outFilterMultimapNmax 1. From here, the same processing was applied as in the main cohort.

### Chromosome transcript ratios and differential gene expression

Transcript ratios for each given chromosome were calculated by summing the raw gene counts of the chromosome of interest and dividing by the sum of gene counts from all other chromosomes [36]. Statistical differences between chromosome transcript ratios in SLE patients and HC patients were measured by the Mann-Whitney U-test and the effect size was calculated by applying Cohen’s distance (Cohen’s D). *P*-values were corrected for multiple cell types, and only results with an False Discovery Rate (FDR) < 0.05 were considered significant.

Differential gene expression was assessed using the edgeR (v3.42.4) R package [68] to compare gene expression in SLE patients and HC patients. Differentially Expressed Genes (DEGs) were filtered by applying a threshold to FDR < 0.05 and no threshold to the logFC. This approach ensured the detection of small but statistically significant changes in X-linked gene expression [69]. DEGs were classified as upregulated or downregulated and further categorized based on consistency across cell types. Genes were considered consistently regulated if they exhibited the same up- or downregulation pattern across all cell types within a defined group (as described above).

To assess the spatial distribution of X-linked DEGs, the genomic distance between these genes was assessed using the closest function of bedtools (v2.31.2). Three comparisons were made for each cell type: distance between closest upregulated genes, distance between closest downregulated genes, distance between closest up- and downregulated genes. The statistical difference between each comparison was evaluated with a Mann-Whitney U-test, with *p*-values corrected per cell type and only results with an FDR < 0.05 were considered significant.

### Correlation and gene set enrichment analyses

Gene expression correlations with X/A-ratio and *XIST* expression were computed using Spearman’s rank correlation. *XIST* correlation analyses were further subdivided into: [1] intracellular correlation, comparing *XIST* expression against remaining genes within the same cell type; [2] intercellular correlation, comparing *XIST* expression from one cell type against remaining genes in all other cell types. Given the number of comparisons, *p*-values were corrected for multiple cell types and multiple genes. In all cases, only genes with an FDR < 0.05 were considered correlated.

GSEA was performed using the gseapy (v1.1.3) Python package for both X/A-correlated genes and *XIST*-correlated genes. Genes were ranked by correlation values and used as input for enrichment analysis. Enrichments were assessed for KEGG Pathways [70]; Biological Processes from Gene Ontology [71, 72] and IFN-signatures [52]. Only statistically significant enrichments were considered (FDR < 0.05).

### SLE classification model through data integration analysis for biomarker discovery using latent components (DIABLO)

An SLE classification model was developed using the DIABLO framework, implemented with the mixOmics (v6.24.0) R package [34]. DIABLO was applied to integrate transcriptomics data across all cell types in the main SLE cohort, selecting the genes from each cell type that best correlate with each other and with patient phenotypes - SLE or HC. Multiple models were tested, either incorporating all expressed genes or restricting features to those on a single chromosome. The final model was selected based on both performance and biological relevance.

The analysis included only female individuals and samples with gene expression data across all cell types. Data normalization and data shape were done as recommended in Singh et al. [73]. Briefly, low-expression genes (CPM < 1 in more than 85% of the patients) and genes with near-zero variance were filtered out. The expression data was then divided into training data and testing data, with a 70/30 balanced split.

Model parameters - design matrix, number of components, and number of variables - were fine-tuned for the best performance. The design matrix was constructed as in Vieira et al. [74] by taking a data-driven approach. Specifically, we used Partial Least Squares (PLS) to assess the correlation between every pair of datasets. Two other design matrixes were also tested, one with 50% of the value from the PLS comparison, and one with 30% of the same value. The number of components was fixed at two. The selection of the number of variables for each cell type and for each component was simplified to shorten the tunning process: the number of variables was always the same for the 27 cell types and for both components. Yet, to get to the number of features that best classify the patients while maintaining biological relevance, a two-step grid search was performed. To ensure reproducibility all searches were cross-validated (CV) using a 5 × 3 arrangement. The best combination of design matrix and number of variables was chosen according to model performance.

Model performance was evaluated using several performance measurements: accuracy, precision, recall, F1, Balanced Error Rate (BER), and the number of variables. The major ranking criterion was F1, with the number of variables being the tiebreaker criteria, so that in cases where two models measure the same F1 score, the model that uses the least variables is considered the best model as it is the simplest. Hence, the chosen model for one chromosome was the best model tested based on the F1 score and model simplicity. Within a specific model, cell type performance was also measured based on the Receiver Operating Characteristic (ROC) curve and the respective Area Under the Receiver Operating Characteristic Curve (AUROC).

Furthermore, the proportion of X/A-ratio correlated genes among the marker genes of each model was statistically tested using Fisher’s Exact Test. Results with a *p*-value < 0.05 were considered significant.

### XIST-interactome and regression models

A set of 107 genes from the XIST-interactome was curated based on the results from Yu et al. [26]. These genes were used to model their relationship with the X/A-ratio in each cell type via linear regression with elastic net regularization, implemented in scikit-learn (v1.4.2). Only differentially expressed *XIST*-interactome genes between SLE and HC were included in the analysis. A range of alpha values was tested, and the optimal parameter was selected via five-fold cross-validation. Model performance was assessed through the R-squared (R2) metric.

### Statistical analyses and code

All analyses were performed in either R (v4.3.0) or python (v3.12.4) using the appropriate packages. Source code for most analyses is available at GitHub: https://github.com/comicsfct/XLupus.

## Supplementary Information

Below is the link to the electronic supplementary material.

Supplementary Table 1

Supplementary Table 2

Supplementary Table 3

Supplementary Table 4

Supplementary Table 5

Supplementary Table 6

Supplementary Table 7

Supplementary Table 8

Supplementary Table 9

Supplementary Table 10

Supplementary Table 11

Supplementary Table 12

Supplementary Table 13

Supplementary Figures

## Data Availability

The data used for this research was obtained by the research group led by Dr. Keishi Fujio and published in “Distinct transcriptome architectures underlying lupus establishment and exacerbation” (10.1016/j.cell.2022.07.021). It is available at the website of the NBDC Human Database (https://humandbs.dbcls.jp/en/) of the Database Center for Life Science (DBCLS) (JGAD000603). All additional data supporting the findings of the study are available within the paper and its Supplementary Tables.
