# Supplementary material for: X-linked transcriptome dysregulation across immune cells in systemic lupus erythematosus
Source: Biol Sex Differ. 2025 Sep 25;16:69. doi: 10.1186/s13293-025-00750-3 (PMC12466074; doi:10.1186/s13293-025-00750-3)
Supplement: Supplementary file 14 — Supplementary Figures [file 13293_2025_750_MOESM14_ESM.pdf]

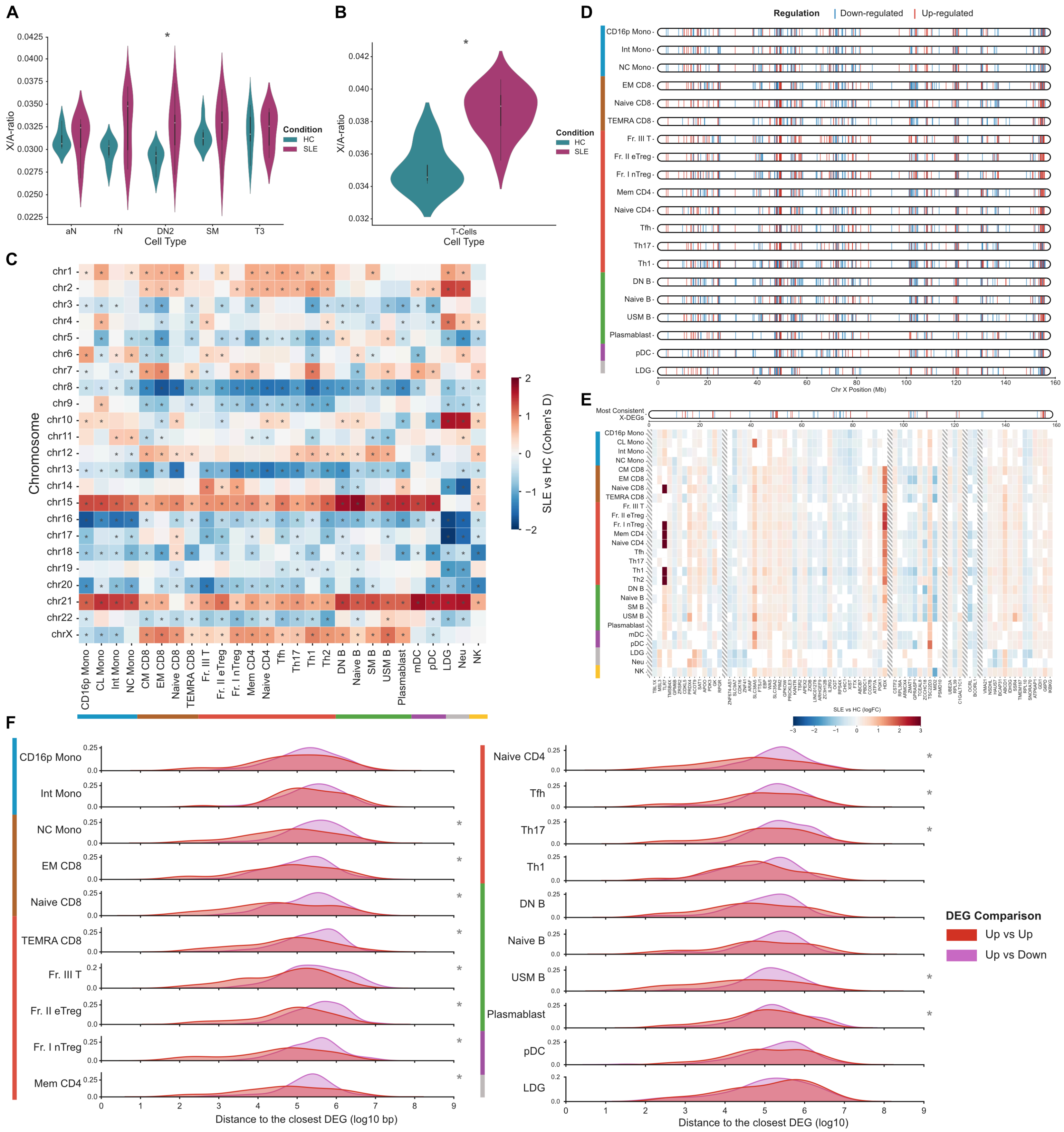

Supplementary Figure 1. X-linked transcriptome dysregulation across immune cells in SLE patients. A) Distribution of X/A-ratio values for SLE and HC samples across the different B-cell subsets from an independent cohort (37). **B)** Distribution of X/A-ratio values for SLE and HC samples in T cells from an independent cohort (38). **C)** Heatmap with Cohen's D effect size of the individual chromosomes transcript ratios between SLE and HC across the different immune cell types. **D)** Genomic localization of up and downregulated genes in SLE along the X chromosome for the immune cell types with X/A-ratio dysregulated in SLE. **E)** Heatmap with transcriptome alterations (logFC) of consistently up and downregulated genes in SLE samples along the X chromosome for the immune cell types with X/A-ratio dysregulated in SLE. Columns with diagonal stripes represent large distances (> 8 Mb) between clusters of dysregulated genes. **F)** Density plot for the genomic distance between the X-linked upregulated genes and the closest upregulated (red) or downregulated gene (pink) for the immune cell types with X/A-ratio dysregulated in SLE. * FDR < 0.05.

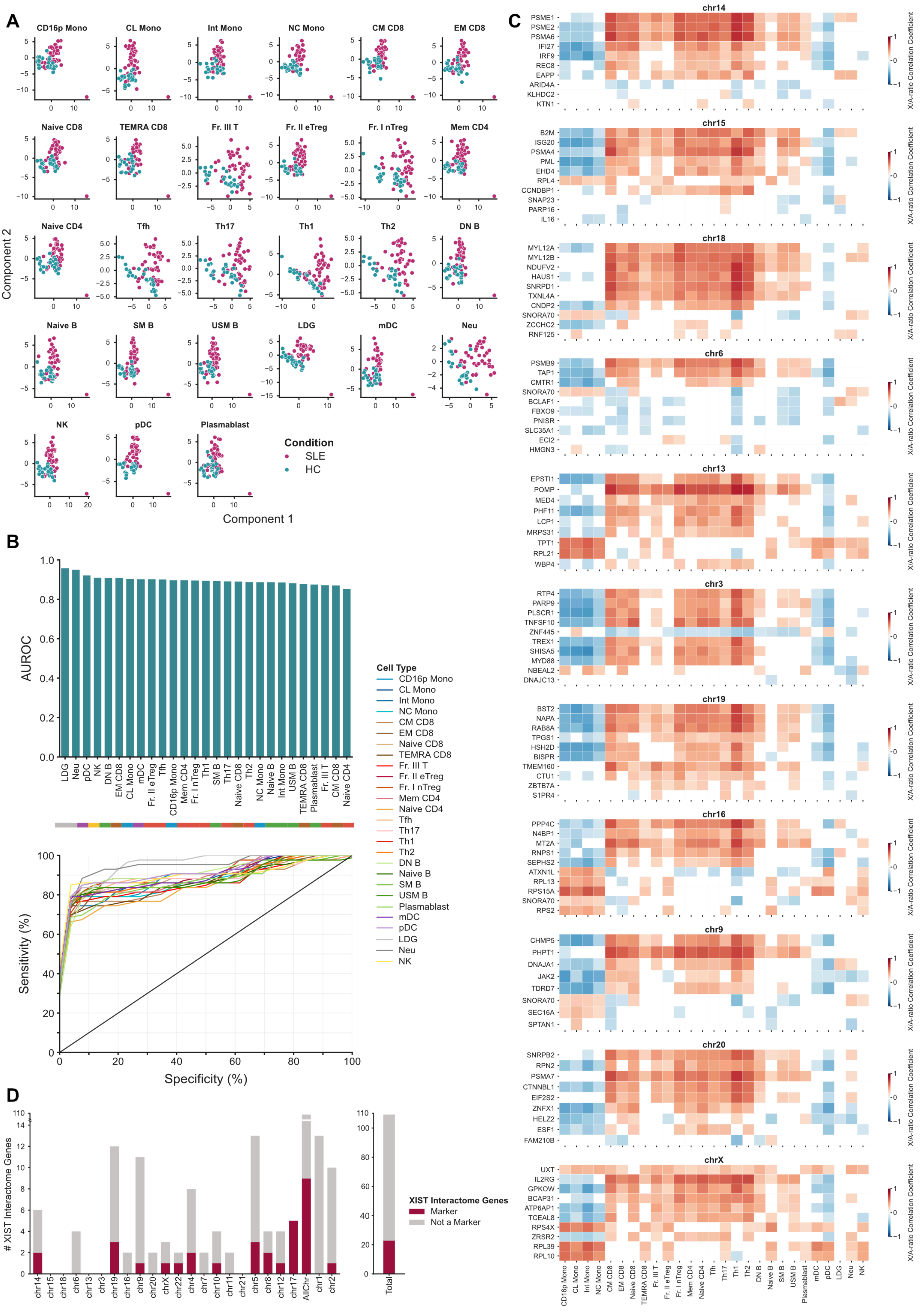
A
CD16p Mono
CL Mono
Int Mono
NC Mono
CM CD8
EM CD8
Naive CD8
TEMRA CD8
Fr. III T
Fr. II eTreg
Fr. I nTreg
Mem CD4
Naive CD4
Tfh
Th17
Th1
Th2
DN B
Naive B
SM B
USM B
LDG
mDC
Neu
NK
pDC
Plasmablast
Component 2
Component 1
Condition
SLE
HC
B
AUROC
LDG
Neu
pDC
NK
DN B
EM CD8
CL Mono
mDC
Fr. II eTreg
Tfh
CD16p Mono
Mem CD4
Fr. I nTreg
Th1
SM B
Th17
Naive CD8
Th2
NC Mono
Naive B
Int Mono
USM B
TEMRA CD8
Plasmablast
Fr. III T
CM CD8
Naive CD4
Sensitivity (%)
Specificity (%)
Cell Type
CD16p Mono
CL Mono
Int Mono
NC Mono
CM CD8
EM CD8
Naive CD8
TEMRA CD8
Fr. III T
Fr. II eTreg
Fr. I nTreg
Mem CD4
Naive CD4
Tfh
Th17
Th1
Th2
DN B
Naive B
SM B
USM B
Plasmablast
mDC
pDC
LDG
Neu
NK
C
chr14
PSME1
PSME2
PSMA6
IFI27
IRF9
REC8
EAPP
ARID4A
KLHDC2
KTN1
chr15
B2M
ISG20
PSMA4
PML
EHD4
RPL4
CCNDBP1
SNAP23
PARP16
IL16
chr18
MYL12A
MYL12B
NDUFV2
HAUS1
SNRPD1
TXNL4A
CNDP2
SNORA70
ZCCHC2
RNF125
chr6
PSMB9
TAP1
CMTR1
SNORA70
BCLAF1
FBXO9
PNISR
SLC35A1
ECI2
HMGN3
chr13
EPSTI1
POMP
MED4
PHF11
LCP1
MRPS31
TPT1
RPL21
WBP4
chr3
RTP4
PARP9
PLSCR1
TNFSF10
ZNF445
TREX1
SHISA5
MYD88
NBEAL2
DNAJC13
chr19
BST2
NAPA
RAB8A
TPGS1
HSH2D
BISPR
TMEM160
CTU1
ZBTB7A
S1PR4
chr16
PPP4C
N4BP1
MT2A
RNPS1
SEPHS2
ATXN1L
RPL13
RPS15A
SNORA70
RPS2
chr9
CHMP5
PHPT1
DNAJA1
JAK2
TDRD7
SNORA70
SEC16A
SPTAN1
chr20
SNRPB2
RPN2
PSMA7
CTNNBL1
EIF2S2
ZNFX1
HELZ2
ESF1
FAM210B
chrX
UXT
IL2RG
GPKOW
BCAP31
ATP6AP1
TCEAL8
RPS4X
ZRSR2
RPL39
RPL10
X/A-ratio Correlation Coefficient
CD16p Mono
CL Mono
Int Mono
NC Mono
CM CD8
EM CD8
Naive CD8
TEMRA CD8
Fr. III T
Fr. II eTreg
Fr. I nTreg
Mem CD4
Naive CD4
Tfh
Th17
Th1
Th2
DN B
Naive B
SM B
USM B
Plasmablast
mDC
pDC
LDG
Neu
NK
D
XIST Interactome Genes
chr14
chr15
chr18
chr6
chr13
chr3
chr19
chr16
chr9
chr20
chrX
chr22
chr4
chr7
chr10
chr11
chr21
chr5
chr8
chr12
chr17
AllChr
chr1
chr2
Total
XIST Interactome Genes
Marker
Not a Marker

Supplementary Figure 2. Integration of Immune Transcriptome profiles for SLE classification. A) SLE and HC samples distribution according to the two components of the X-model for each immune cell type. **B)** X-model cell performance according to the AUROC values and ROC curves for each cell type. **C)** Heatmap with correlation coefficients between X/A-ratio and the markers of the individual chromosome-based models (FDR < 0.05). **D)** Number of XIST-interactome genes selected as markers for each individual chromosome and all chromosome models.

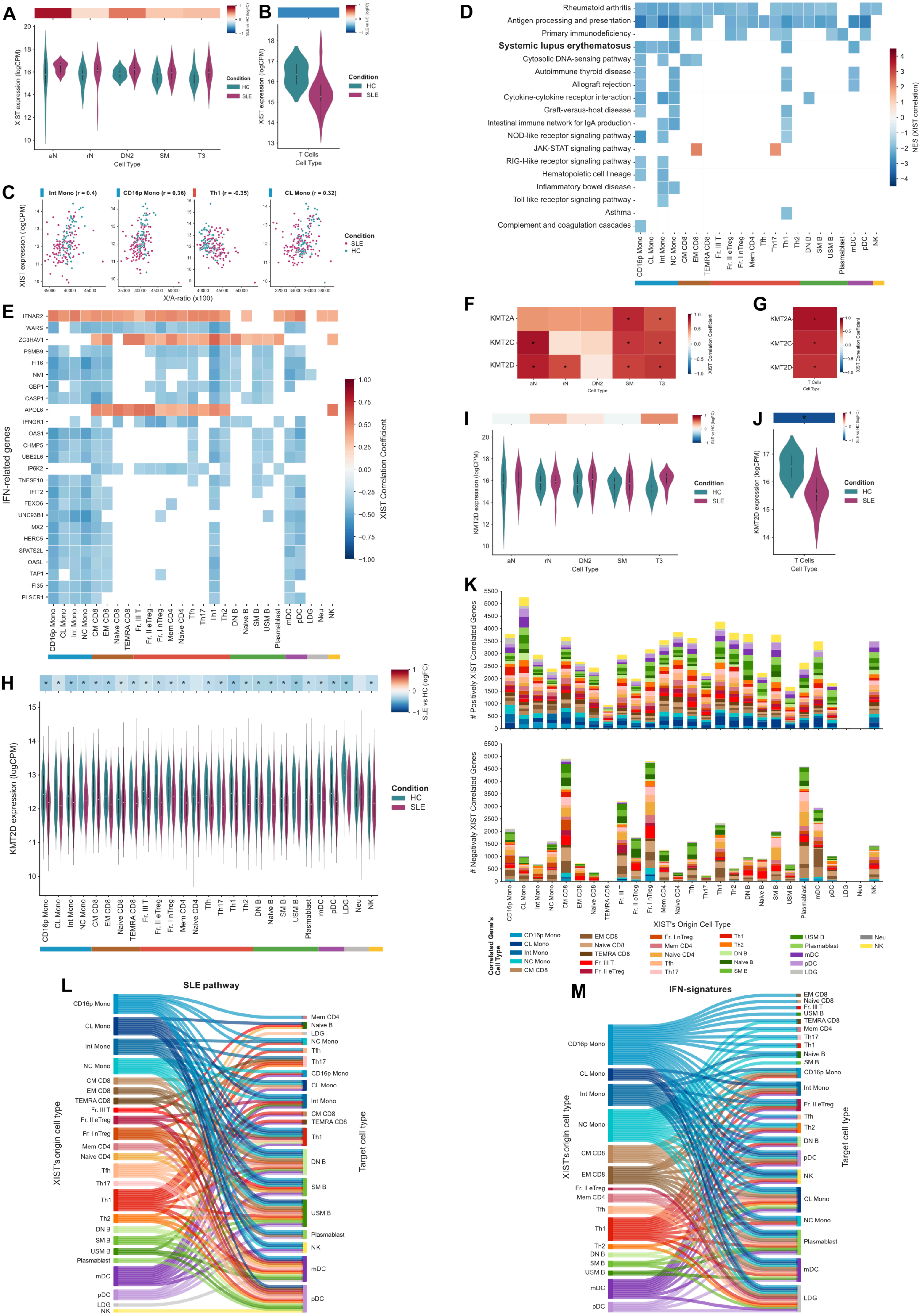

A
XIST expression (logCPM)
Cell Type
aN
rN
DN2
SM
T3
Condition
HC
SLE
SLE vs HC (logFC)
B
T Cells
C
Int Mono (r = 0.4)
CD16p Mono (r = 0.36)
Th1 (r = -0.35)
CL Mono (r = 0.32)
X/A-ratio (x100)
D
Rheumatoid arthritis
Antigen processing and presentation
Primary immunodeficiency
Systemic lupus erythematosus
Cytosolic DNA-sensing pathway
Autoimmune thyroid disease
Allograft rejection
Cytokine-cytokine receptor interaction
Graft-versus-host disease
Intestinal immune network for IgA production
NOD-like receptor signaling pathway
JAK-STAT signaling pathway
RIG-I-like receptor signaling pathway
Hematopoietic cell lineage
Inflammatory bowel disease
Toll-like receptor signaling pathway
Asthma
Complement and coagulation cascades
NES (XIST correlation)
E
IFN-related genes
IFNAR2
WARS
ZC3HAV1
PSMB9
IFI16
NMI
GBP1
CASP1
APOL6
IFNGR1
OAS1
CHMP5
UBE2L6
IP6K2
TNFSF10
IFIT2
FBXO6
UNC93B1
MX2
HERC5
SPATS2L
OASL
TAP1
IFI35
PLSCR1
XIST Correlation Coefficient
CD16p Mono
CL Mono
Int Mono
NC Mono
CM CD8
EM CD8
Naive CD8
TEMRA CD8
Fr. III T
Fr. II eTreg
Fr. I nTreg
Mem CD4
Naive CD4
Tfh
Th17
Th1
Th2
DN B
Naive B
SM B
USM B
Plasmablast
mDC
pDC
LDG
Neu
NK
F
KMT2A
KMT2C
KMT2D
XIST Correlation Coefficient
G
H
KMT2D expression (logCPM)
I
J
K
Positively XIST Correlated Genes
Negatively XIST Correlated Genes
XIST's Origin Cell Type
Correlated Gene's Cell Type
L
SLE pathway
XIST's origin cell type
Target cell type
M
IFN-signatures

Supplementary Figure 3. *XIST* lncRNA dysregulation across immune cells in SLE patients. A) Distribution of *XIST* expression levels for SLE and HC samples across the different B cell subsets from an independent cohort (37). Top heatmap indicates the fold-change effect size (logFC) and statistical significance (* FDR < 0.05). **B)** Distribution of *XIST* expression levels for SLE and HC in T cells from an independent cohort (38). Top heatmap indicates the fold-change effect size (logFC) and statistical significance (* FDR < 0.05). **C)** Correlation plots and coefficients for the cell types with the strongest correlation between *XIST* expression levels and X/A-ratio. **D)** Heatmap with Normalized Enrichment Scores (NES) for Gene Set Enrichment Analysis of immune-related KEGG pathways ranking genes according to the *XIST* correlation coefficient (FDR < 0.05). **E)** Heatmap with correlation coefficients between *XIST* expression levels and the and the IFN-related genes (FDR < 0.05). **F)** Heatmap with correlation coefficients between *XIST* and histone lysine methyltransferases of H3K4 across the different B cell subsets from an independent cohort (37) (* FDR < 0.05). **G)** Heatmap with correlation coefficients between *XIST* and histone lysine methyltransferases of H3K4 in T cells from an independent cohort (38) (* FDR < 0.05). **H)** Distribution of *KMT2D* expression levels for SLE and HC samples across the different immune cell types. Top heatmap indicates the fold-change effect size (logFC) and statistical significance (* FDR < 0.05). **I)** Distribution of *KMT2D* expression levels for SLE and HC samples across the different B cell subsets from an independent cohort (37). Top heatmap indicates the fold-change effect size (logFC) and statistical significance (* FDR < 0.05). **J)** Distribution of *KMT2D* expression levels for SLE and HC samples in T cells from an independent cohort (38). Top heatmap indicates the fold-change effect size (logFC) and statistical significance (* FDR < 0.05). **K)** Number of the significant intercellular positive (top plot) and negative (bottom plot) correlations (FDR and |r| > 0.5) between *XIST* expression levels of each cell type (x-axis) and all other genes in other cell types (bar colors). **L)** Sankey plot with the intercellular correlations where *XIST* expression in the origin cell type is correlated with the expression of SLE-associated genes in the target cell type (FDR < 0.05). **M)** Sankey plot with the intercellular correlations where *XIST* expression in the origin cell type is correlated with the expression of IFN-signatures in the target cell type (FDR < 0.01 and NES < -3).

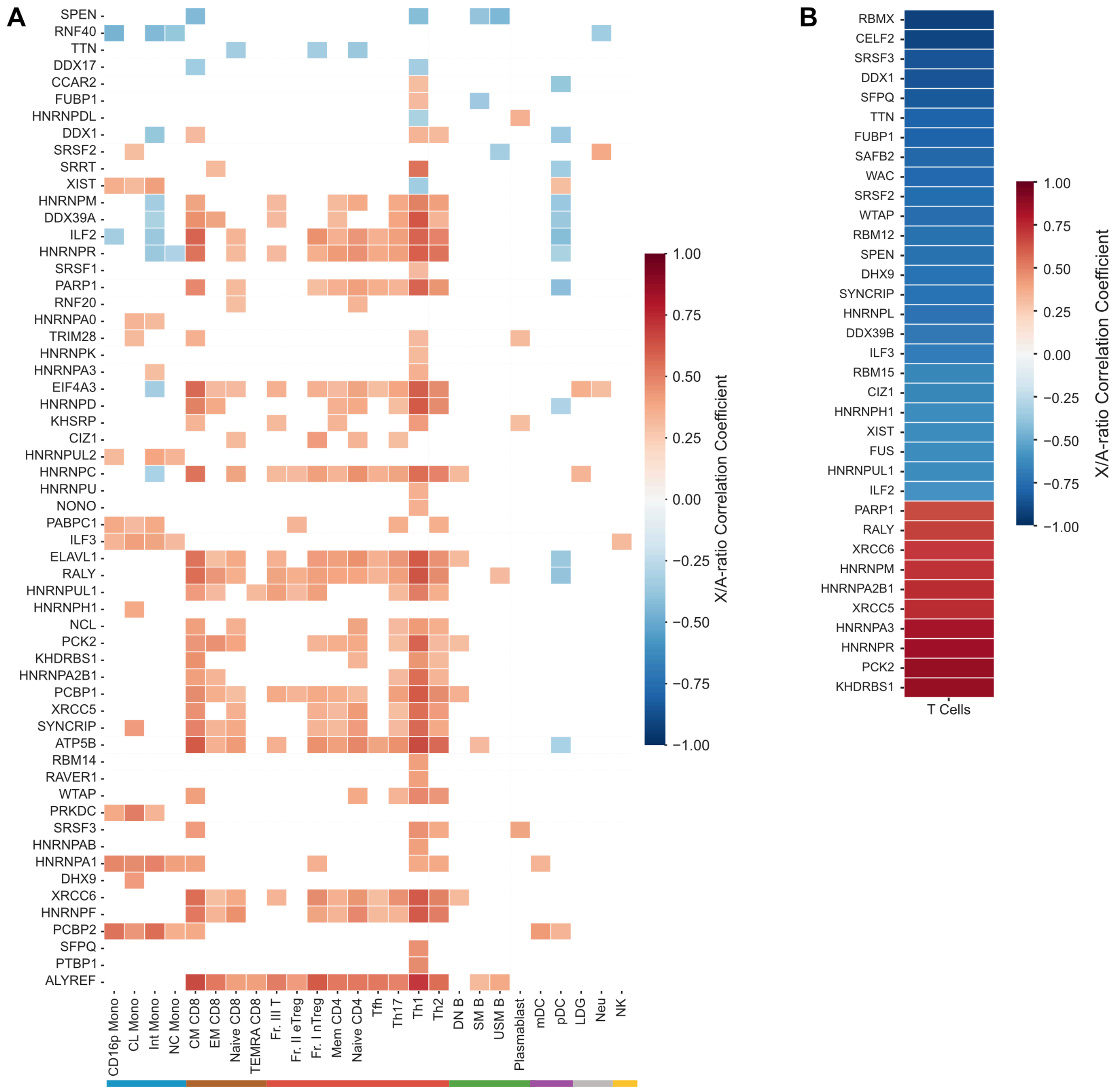

Supplementary Figure 4. XIST-interactome dysregulation across immune cells in SLE patients. A) Heatmap with correlation coefficients between X/A-ratio and gene expression levels of XIST-interactome (FDR < 0.05). **B)** Heatmap with correlation coefficients between X/A-ratio and gene expression levels of XIST-interactome (FDR < 0.05) in T cells of an independent cohort (38).
